# Bisphenol A Alters the Expression of Genes Involved in Lipogenesis, Inflammation, and Oxidative Stress in the Liver of Adult Zebrafish

**DOI:** 10.3390/ph18111765

**Published:** 2025-11-20

**Authors:** Eronides Anathan de Heberle Salau, Daniela Diglio, Giuliano Rizzotto Guimarães, Orlando Vieira Furtado-Filho, Marilene Porawski

**Affiliations:** 1 Graduate Program in Hepatology, Federal University of Health Sciences of Porto Alegre (UFCSPA), Porto Alegre 90050-170, Brazil; marilenep@ufcspa.edu.br; 2 Graduate Program in Biosciences, Federal University of Health Sciences of Porto Alegre (UFCSPA), Porto Alegre 90050-170, Brazil; nutridanni@gmail.com (D.D.); orlandovff@gmail.com (O.V.F.-F.); 3 Pathology Laboratory, Federal University of Health Sciences of Porto Alegre (UFCSPA), Porto Alegre 90050-170, Brazil; giulianog@ufcspa.edu.br

**Keywords:** MASLD, steatosis, liver disease, bisphenol-A, zebrafish, oxidative stress

## Abstract

**Background:** Bisphenol A (BPA) is a widespread environmental endocrine disruptor associated with metabolic dysfunction-associated steatotic liver disease (MASLD). However, its short-term effects at low, environmentally relevant concentrations are still poorly understood. **Methods:** Adult zebrafish were exposed to 5, 20, or 100 µg/L BPA for 48 h, 7, or 14 days in a pilot test. The lowest effective condition (20 µg/L for 7 days) was selected for a complete experiment. Fish were divided into two groups: control and BPA-exposed (*n* = 50/group). After exposure, livers were collected for histological (HE, Oil Red O, Nile Red) and molecular (RT-qPCR) analyses. **Results:** Exposure to 20 µg/L BPA for 7 days induced moderate to severe hepatic steatosis, characterized by vacuolization, hepatocyte ballooning, and lipid accumulation. Gene expression analysis showed upregulation of fasn (fatty acid synthase), *acc1* (acetyl-CoA carboxylase 1), *srebp-1c* (sterol regulatory element-binding protein 1c), *nfkb* (nuclear factor kappa B), il-6 (interleukin-6), *gpx1* (glutathione peroxidase 1), *sod* (superoxide dismutase), *cyp1a* (cytochrome P450 1A), and *cyp2ad2* (cytochrome P450 2AD2), while *adipor2* (adiponectin receptor 2) and *gpx4* (glutathione peroxidase 4) were downregulated (decreased activity). **Conclusions:** Short-term exposure to a low, environmentally relevant concentration of BPA was sufficient to trigger hepatic steatosis in zebrafish. These effects were associated with enhanced lipogenesis, inflammation, oxidative imbalance, and altered xenobiotic metabolism, suggesting that even brief, low-dose BPA exposure may contribute to early events in MASLD pathogenesis.

## 1. Introduction

Metabolic Dysfunction-Associated Steatotic Liver Disease (MASLD), formerly known as Non-alcoholic Fatty Liver Disease (NAFLD), is the most common form of chronic liver disease, resulting from lipid metabolism dysfunctions and characterized by the accumulation of lipids in liver cells [1,2]. MASLD encompasses a spectrum of liver injuries, ranging from simple steatosis to steatohepatitis, fibrosis, and cirrhosis [3]. Bisphenol A (BPA) is ubiquitous in the environment, as it is a component of many consumer products, including household items, industrial goods, and plastics, and can be found in all environmental matrices, such as water, soil, and air [4]. BPA is part of the micropollutants classified as endocrine disruptors (EDs), which are exogenous agents that interfere with the synthesis, secretion, transport, metabolism, or binding activity, or removal of natural hormones from the bloodstream, which are essential for homeostasis, reproduction, and development processes [5,6] Previous studies demonstrated that BPA acts as an obesogenic compound that disrupts lipid metabolism. Exposure to BPA has been associated with both the initiation and progression of MASLD [7,8]. Moreover, experimental studies in rats have shown that even short-term BPA exposure can induce early hepatic steatotic changes, supporting its role as an initiator of metabolic liver dysfunction [9,10]. In zebrafish, lower BPA concentrations have been reported to increase triglyceride storage and promote fatty acid synthesis, whereas higher concentrations enhance de novo lipogenesis and cholesterologenesis, further indicating that BPA contributes to hepatic lipid accumulation and obesity development [11]. Previous studies investigating Bisphenol A (BPA)-induced toxicity in zebrafish have generally employed higher concentrations and longer exposure periods, often exceeding environmentally relevant levels. For instance, adult zebrafish exposed to 100 µg/L of BPA for 60 days exhibited significant hepatic lipid accumulation, oxidative stress, and alterations in lipid metabolism [12]. Similarly, another study exposed different groups to concentrations ranging from 100 µg/L to 30 mg/L for periods varying between 96 h and 3 months, resulting in developmental toxicity, apoptosis, and hematopoietic dysfunction in embryos and larvae [13]. In addition, short-term exposure (3 days) to high concentrations of BPA (1–5 mg/L) in zebrafish larvae induced hepatotoxicity characterized by hepatocyte vacuolization, oxidative stress, and increased apoptosis [14].

In contrast, the present study focuses on short-term exposure (7 days) to a low and environmentally realistic concentration (20 µg/L), aiming to identify early molecular and histological events characteristic of MASLD. By comparing low-dose, short-term exposure with previous studies that used higher concentrations or longer exposure periods, our findings provide new insights into how even brief contact with environmentally relevant levels of BPA can trigger hepatic steatosis and disrupt lipid metabolism.

The zebrafish is an excellent model for studying metabolic dysfunctions due to its conserved functions, including appetite regulation, insulin regulation, and lipid storage [15,16]. The liver is the main organ involved in the biotransformation of xenobiotics and the primary site of expression for cytochrome P450 (*cyp450*) enzymes. Zebrafish liver cells express *cyp450* enzymes that metabolize xenobiotic compounds via the same pathway as mammalian hepatocytes [17,18]. The liver also plays a role in lipid metabolism, and hepatic lipogenesis is transcriptionally regulated by the sterol regulatory element-binding protein 1c (*srebp-1c*), Acetyl-CoA carboxylase 1 (*acc1*), and fatty acid synthase (*fas*) [19]. The gene expression of *srebp-1c* modulates the expression of *acc1* and *fas* genes, which directly contribute to de novo lipogenesis in the liver [20,21]. The nuclear factor kappa B (*nf-κb*) transcription factor is crucial in mediating and regulating the expression of several inflammation-related genes, including interleukin-6 (*il-6*). Initially inactive, *nf-κb* resides in the cytoplasm. Upon phosphorylation and activation, it migrates to the nucleus, where it associates with promoters of specific genes, exerting positive control over the transcription of cytokines and chemokines [22]. The adiponectin receptor 2 (*adipor2*) is one of the three adiponectin receptors, with adiponectin being an adipokine produced by adipose tissue that may play a crucial role in protecting against insulin resistance/diabetes and atherosclerosis [23,24]. Glutathione peroxidase 1 (*gpx1*) influences thiol redox status and balances required and harmful cellular oxidants. An accumulation of intracellular oxidants can promote redox-mediated signaling events and activate pro-inflammatory pathways mediated by redox-sensitive transcription factors, such as *nf-κb* [25]. Glutathione peroxidase (*gpx4*) is a unique antioxidant enzyme within cells, responsible for directly reducing peroxidized phospholipids that form in the cellular membrane [26]. Superoxide dismutase (*sod*) catalyzes the conversion of superoxide free radicals, inhibits reactive oxygen species (ROS) production in the liver, and reduces intrahepatic inflammation [27,28].

This study utilized zebrafish as an experimental model to examine the hepatic effects of BPA, specifically addressing alterations in lipid metabolism, inflammatory responses, xenobiotic biotransformation, and oxidative stress. Additionally, the research established the minimum duration and concentration required to induce hepatic steatosis in zebrafish.

## 2. Results

### 2.1. Histological Analysis of the Pilot Test

**Figure 1:**
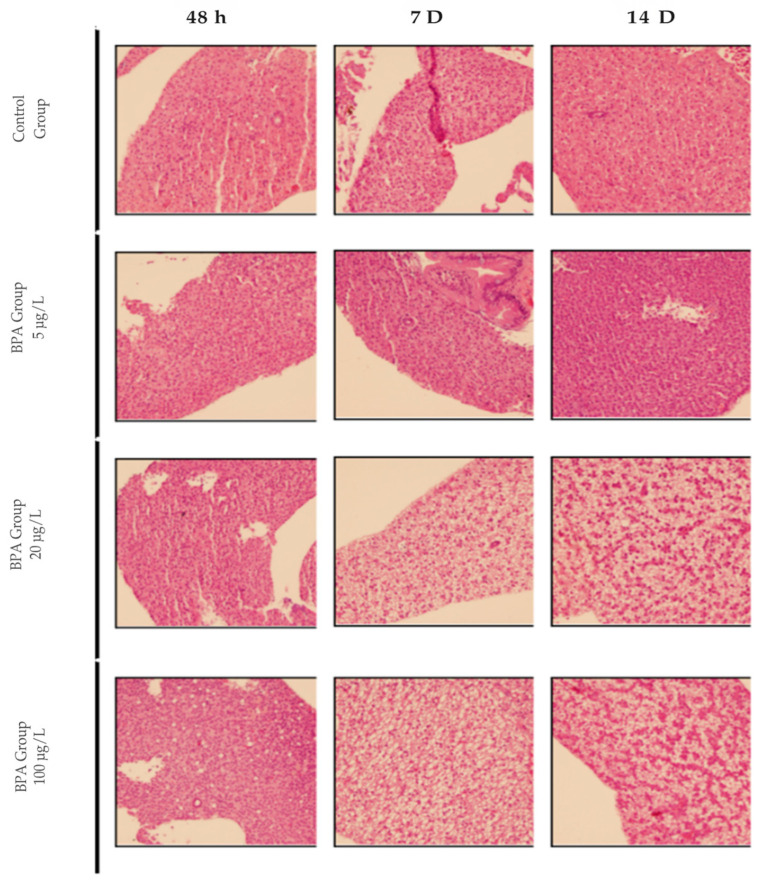
Photomicrograph of adult zebrafish liver with Hematoxylin and Eosin (HE) staining. Representative images of control animals and those exposed to concentrations of 5, 20 and 100 µg/L of BPA at 48 h, 7 D or 14 D. 20× magnification.

Histological analysis of liver samples from pilot test animals revealed that exposure to bisfenol A at concentrations used in the study for up to 48 h did not induce hepatic steatosis. After 7 and 14 days, animals exposed to 20 and 100 µg/L concentrations showed moderate to severe steatosis. The concentration of 5 µg/L did not induce steatosis at any of the times evaluated in this study. The pilot test results indicated that the lowest concentration and shortest time capable of inducing moderate to severe hepatic steatosis in adult zebrafish were 20 µg/L for 7 days (Figure 1). No hepatic changes were observed in the control group.

### 2.2. Histological and Histochemical Analysis of Zebrafish Liver Exposed to 20 µg/L of BPA

**Figure 2:**
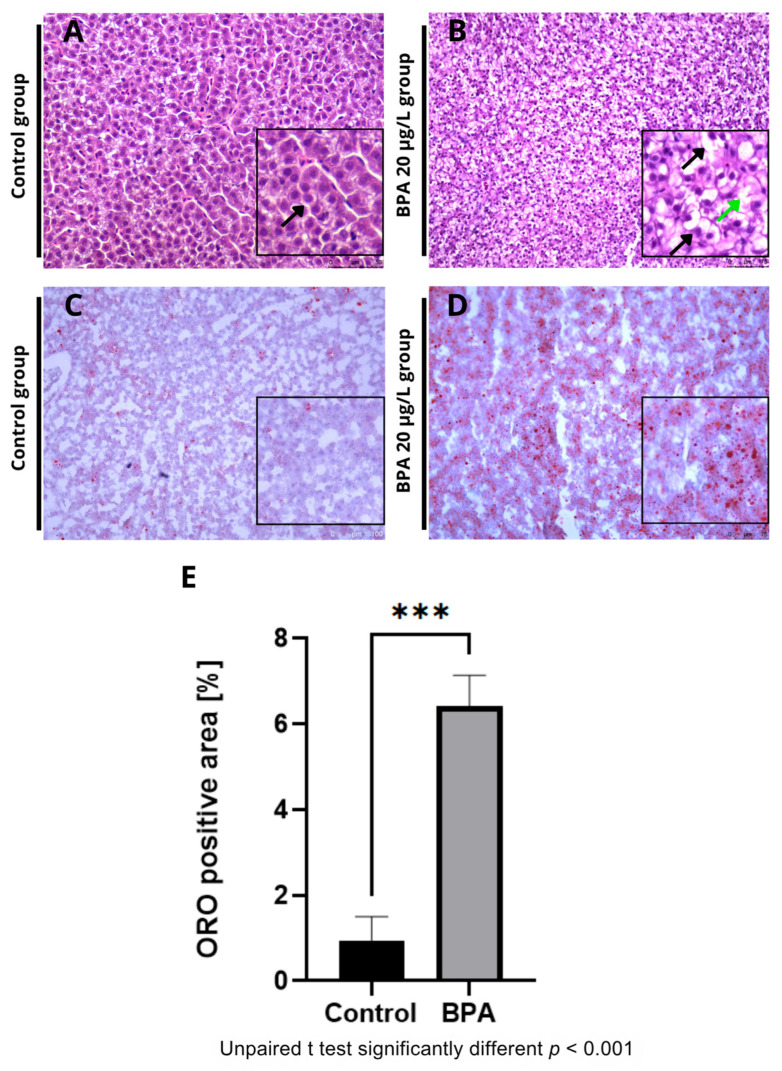
Photomicrographs of adult zebrafish livers stained with Hematoxylin and Eosin (HE) (**A**,**B**) and Oil Red O (**C**,**D**) at 20× magnification. (**A**) Control group showing preserved liver parenchyma and cells with a central, spherical nucleus (black arrow). (**B**) BPA 20 µg/L group showing enlarged hepatocytes containing cytoplasmic vacuoles, with nuclei displaced toward the cell periphery and evidence of ballooning (green arrow). (**C**) Control group showing no morphological changes and fat droplets dispersed within the liver parenchyma. (**D**) BPA 20 µg/L group showing lipid droplets stained red (black arrow) in detail. (**E**) Quantification of the fat deposition area (Oil Red O–positive area). A significant difference (*** *p* ≤ 0.001) was observed compared with the control group (*n* = 3). Asterisks indicate a significant difference between the exposed and control groups (Student’s *t*-test; *** *p* ≤ 0.001). Image analysis was performed using ImageJ software, version 1.54a (National Institutes of Health, Bethesda, MD, USA).

**Figure 3:**
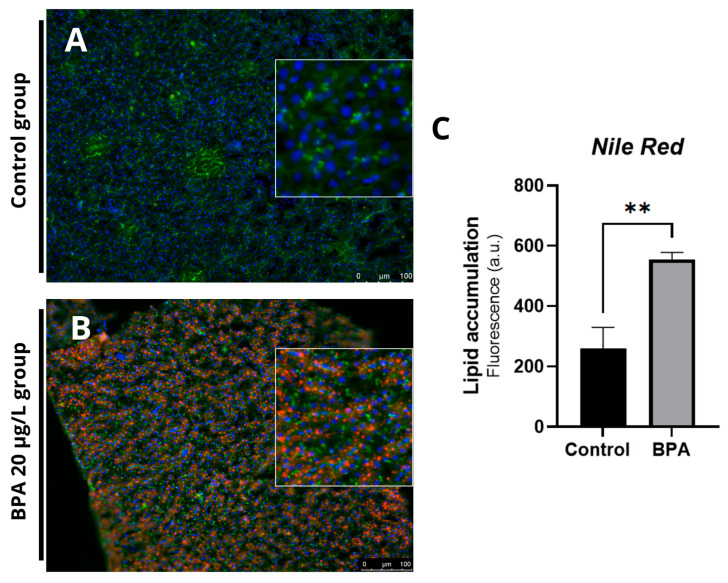
Photomicrograph of adult zebrafish livers stained with Nile Red and observed under a fluorescence microscope at 20× magnification (green staining marks the cytoplasm, blue marks the nucleus, and red marks the lipids). (**A**) Control group showing no morphological changes and well-preserved cytoplasm. (**B**) Presence of lipid droplets indicating fat accumulation and cytoplasmic alteration. (**C**) Lipid content estimated by nile red staining in liver homogenates (20 mg) from zebrafish exposed to BPA and from control livers (*n* = 3). Results are expressed as arbitrary fluorescence units. Asterisks indicate a significant difference between the exposed and control groups (Student’s *t*-test; ** *p* ≤ 0.01).

Histological sections revealed vacuoles and ballooning in the hepatocytes of fish exposed to 20 µg/L BPA (Figure 2A) compared to the control group (Figure 2B). No hepatic changes were observed in the control group. Additionally, Oil Red O staining revealed an accumulation of fat in the hepatic parenchyma in the BPA groups (Figure 2C) compared to the control group (Figure 2D). Quantification of the fat deposition area showed a significant difference (*p* ≤ 0.001) in the positive fat area compared to the control group (Figure 2E). Histochemical staining with Nile Red confirmed the accumulation of lipids in the liver of animals exposed to Bisphenol-A (Figure 3A,B). Furthermore, the spectrophotometric assay for neutral lipid quantification demonstrated a significantly higher accumulation of lipids in the livers of BPA-exposed zebrafish compared to controls (*p* ≤ 0.01), corroborating the histological and histochemical findings (Figure 3C). Histological and lipid quantification data are shown in Supplementary Table S2.

### 2.3. Expression of Genes Related to Lipid Metabolism

**Figure 4:**
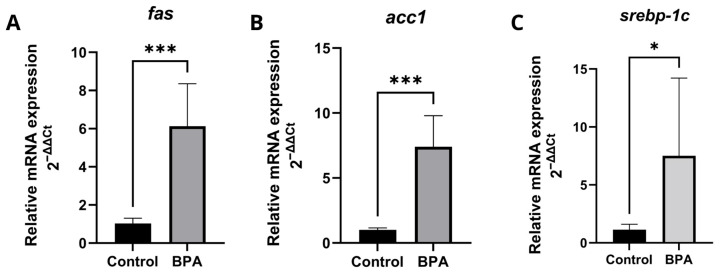
Expression profiles of genes involved in fatty acid synthesis in zebrafish exposed to BPA. Five pools, each consisting of three livers per experimental group, were analyzed in duplicate (*n* = 5). Data are expressed as mean ± standard deviation. Asterisks indicate significant differences between the exposed and control groups (Student’s *t*-test * *p* ≤ 0.05; *** *p* ≤ 0.001). *fasn* (fatty acid synthase) (**A**); *acc1* (acetyl-CoA carboxylase 1) (**B**); *srebp-1c* (sterol regulatory element-binding transcription factor 1) (**C**).

The transcriptional levels of genes related to lipid metabolism were detected by RT-qPCR (Figure 4). In zebrafish liver, the genes: fatty acid synthase (*fasn*) (Figure 4A), acetyl-CoA carboxylase 1 (*acc1*) (Figure 4B), sterol regulatory element-binding transcription factor 1 (*srebp-1c*) (Figure 4C), had significantly increased expression levels in the liver of zebrafish exposed to 20 μg/L BPA. Fas and acc1 actively participate in the synthesis of fatty acids and had a substantially more significant difference compared to *srebp-1c*. Detailed qPCR fold-change values and statistical results are provided in Supplementary Table S1.

### 2.4. Expression of Genes Related to Immune Response, Inflammation, and Metabolism

**Figure 5:**
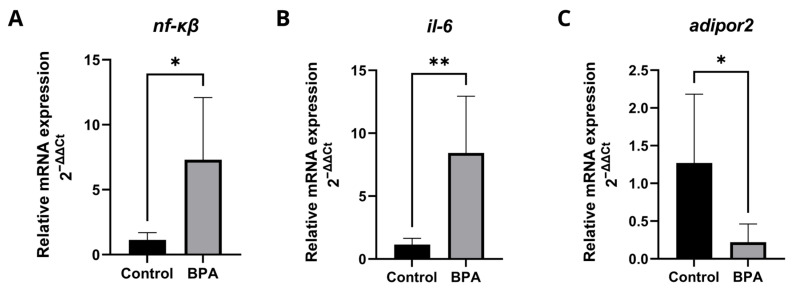
Transcription profiles of genes involved in the inflammatory response. Five pools, each consisting of three livers per experimental group, were analyzed in duplicate (*n* = 5). Data are expressed as mean ± standard deviation. Asterisks indicate significant differences between the exposed and control groups (Student’s *t*-test * *p* ≤ 0.05; ** *p* ≤ 0.01). *nfκb* (nuclear factor kappa B) (**A**); *il-6* (interleukin 6) (**B**); *adipor2* (adiponectin receptor 2) (**C**).

The transcriptional levels of genes involved in the inflammatory process (Figure 5) showed that BPA induced an inflammatory response and inhibited adiponectin receptor, which, in addition to being related to insulin sensitivity and lipid metabolism, has anti-inflammatory properties in the liver. The expression levels of the genes *nf-κβ* (Figure 5A) and *il-6* (Figure 5B) increased significantly, while *adipor2* (Figure 5C) expression decreased. Detailed qPCR fold-change values and statistical results are provided in Supplementary Table S1.

### 2.5. Expression of Genes Related to Oxidative Stress and Biotransformation of Xenobiotics

**Figure 6:**
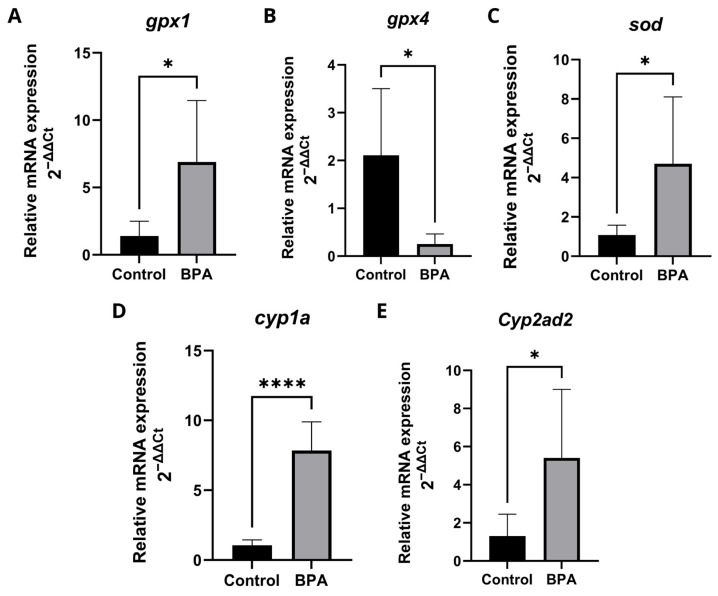
Transcription profiles of genes involved in the antioxidant defense mechanism. Five pools, each consisting of three livers per experimental group, were analyzed in duplicate (*n* = 5). Data are expressed as mean ± standard deviation. Asterisks indicate significant differences between the exposed and control groups (Student’s *t*-test * *p* ≤ 0.05; **** *p* ≤ 0.0001). *gpx1* (glutathione peroxidase 1) (**A**); *gpx4* (glutathione peroxidase 4) (**B**); *sod* (superoxide dismutase) (**C**); *cyp1a* (cytochrome P450 1A) (**D**); *cyp2ad2* (cytochrome P450 2AD2) (**E**).

The transcriptional levels of genes involved in oxidative stress and xenobiotic biotransformation were detected by RT-qPCR (Figure 6). In the liver of zebrafish, the gene glutathione peroxidase 1 (*gpx1*) (Figure 6A) and superoxide dismutase (*sod*) (Figure 6C) had their expression levels significantly increased, glutathione peroxidase 4 (*gpx4*) (Figure 6B) had its expression levels significantly reduced. The Cytochrome P450 1A (*cyp1a*) (Figure 6C) and Cytochrome P4502Ad2 (*cyp2ad2*) (Figure 6D) genes had significantly increased expression levels in the liver of zebrafish exposed to 20 μg/L BPA. Detailed qPCR fold-change values and statistical results are provided in Supplementary Table S1.

## 3. Discussion

By 2030, metabolic dysfunction-associated steatotic liver disease (MASLD) is expected to be the leading cause of chronic liver disease in adults and children and may become the primary indication for liver transplantation [29]. MASLD is a spectrum of diseases ranging from hepatic steatosis to steatohepatitis associated with metabolic dysfunction (MASH) with a risk of progression to cirrhosis and hepatocellular carcinoma (HCC) [30]. It is part of the metabolic syndrome and, consequently, is associated with obesity, insulin resistance (IR), and type 2 diabetes mellitus (T2DM) [31]. Bisphenol A (BPA) is widely disseminated in the environment [32]. Previous studies have demonstrated that zebrafish exposed to BPA exhibit steatosis to varying degrees, depending on the concentration, characterized by severe microvesicular fatty changes, hepatocyte ballooning, inflammatory cell infiltration, and pyknotic nuclei [33]. In the present study, adult zebrafish exposed to subchronic and environmentally realistic concentrations of BPA (20 µg/L) exhibited histopathological features compatible with hepatic steatosis, including hepatocellular vacuolation and lipid droplet accumulation. Lipid accumulation in hepatocytes impairs lipid metabolism by altering fatty acid synthesis, transport, and degradation [34]. These findings are consistent with data from animal and human models, in which BPA exposure has been associated with hepatic steatosis and dysregulation of lipogenic and metabolic pathways [35]. The presence of hepatic steatosis is related to insulin resistance and chronic inflammation, increasing the risk of progressive liver disease with fibrosis, cirrhosis, and increased risk of HCC [36,37]. Evidence suggests that BPA may trigger lipid accumulation by disrupting the transcriptional regulation of genes that control fatty acid synthesis and oxidation, thereby disturbing hepatic lipid homeostasis. In rats exposed to BPA, key genes involved in fatty acid β-oxidation, including peroxisome proliferator-activated receptor alpha (pparα) and carnitine palmitoyltransferase 1α (cpt1α), are downregulated, while lipogenic genes such as sterol regulatory element-binding protein-1 (srebp-1), acetyl-CoA carboxylase 1 (acc1), fatty acid synthase (fas), and stearoyl-CoA desaturase 1 (scd-1) are upregulated [38]. The transcription factor SREBP-1c acts as a central regulator of hepatic lipid metabolism, playing a pivotal role in the pathogenesis of steatohepatitis, obesity, and even hepatocellular carcinoma [39]. The expression of the *srebp-1c* gene plays a fundamental role in the pathogenesis of steatohepatitis, obesity, and cancer [40]. Overexpression of *srebp-1c* in adult mice caused a 26-fold increase in fatty acid synthesis and fat deposition [41]. SREBP-1c directly regulates the expression of the main lipogenic enzymes ACC1 and FAS [42]. In our study, BPA exposure significantly upregulated srebp-1c, acc1, and fas in the zebrafish liver, confirming the activation of the lipogenic program also observed in mammalian models. In addition to stimulating lipogenesis, BPA appears to repress lipid β-oxidation by downregulating pparα and upregulating the nuclear receptor pparγ, further exacerbating lipid storage and metabolic imbalance [43]. The resulting steatotic environment is known to enhance the activation of the nuclear transcription factor kappa-B (NF-κB) through IKKβ-mediated degradation of its inhibitor, triggering a pro-inflammatory cascade characterized by elevated expression of interleukin-6 (IL-6) [43,44]. Consistent with this mechanism, our results demonstrated a significant increase in nf-κb and il-6 expression in the zebrafish liver, indicating that BPA exposure promotes hepatic inflammation via NF-κB-dependent signaling.

Our findings showed a downregulation in *adipor2* expression in the liver of zebrafish exposed to BPA, suggesting a direct interference of this endocrine disruptor with the adiponectin signaling pathway. Adiponectin plays a central role in regulating energy metabolism and protecting against obesity and metabolic syndrome. Adipor2, predominantly expressed in the liver, mediates many of its beneficial effects, including ppar-α activation, increased fatty acid β-oxidation, and decreased inflammation and oxidative stress [45]. BPA, recognized as a risk factor for the development of obesity and metabolic syndrome, acts as a hormonal disruptor, altering nuclear receptors and hepatic metabolic pathways [46]. Thus, the decrease in AdipoR2 expression observed in our study may represent one of the mechanisms by which BPA impairs hepatic metabolism. In murine models, AdipoR2 deficiency leads to hyperinsulinemia, reduced PPAR-α activation, and increased oxidative stress and hepatic inflammation [47], whereas its overexpression improves glucose homeostasis, insulin sensitivity, and reduces hepatic steatosis [48]. These findings reinforce the notion that adiponectin/AdipoR2 signaling plays a protective role against metabolic and inflammatory disturbances.

Therefore, BPA-induced downregulation of AdipoR2 may compromise the hepatic defense mechanisms mediated by adiponectin, promoting lipid accumulation, oxidative stress, and inflammatory processes. The modulation of this pathway may thus represent an important protective target against BPA-induced hepatotoxicity, supporting the notion that the integrity of adiponectin signaling is crucial for hepatic homeostasis and regeneration [49]. This evidence suggests a possible pathway through which BPA exerts its obesogenic and hepatotoxic effects, highlighting adiponectin signaling as a key target disrupted by this compound.

There are clear correlations between the activities of certain CYPs and the toxicity of certain chemical substances, either through the activation or deactivation of these substances. Research with knockout mice has indicated that CYPs mediate the harmful effects of various environmental chemicals, drugs, and chemical compounds [50]. *cyp1a* is a key phase I xenobiotic metabolizing enzyme that plays a distinct role in the metabolic activation or clearance of various chemical compounds and endogenous substances and in the adaptive response mechanism against the adverse effects of oxidative stress [51,52]. There is a paucity of information on the expression levels of CYP450 genes in the liver of fish exposed to BPA. In the context of our study, we observed a significant increase in the expression of two CYP450 subunits, *cyp1a* and *cyp2ad2*, which suggests that the xenobiotic biotransformation mechanism is responding compensatorily to subchronic BPA exposure and oxidative stress in the liver of zebrafish. Our findings reveal a significant decrease in the *gpx4*, a gene linked to lipid peroxidation, in the liver of zebrafish exposed to BPA. *gpx4* inhibition increases lipid peroxidation [53]. This result indicates impairment in lipid peroxidase activity, which can lead to the accumulation of lipid peroxides. On the other hand, there was an increase in the expression of the *gpx1* and *sod* genes. *sod* is an essential antioxidant enzyme in the body’s defense against free radicals and reactive oxygen species, and *gpx1* reduces toxic peroxides in the body, including hydrogen peroxide, cholesterol peroxide, and lipid peroxide, modulates the balance between necessary and harmful levels of H_2_O_2_ and protects against the progression and development of many diseases [54]. Thus, our findings suggest that BPA inhibits the expression of the *gpx4* gene in the liver of zebrafish, resulting in the accumulation of lipid peroxides that stimulate the expression of the *gpx1* and sod genes, highlighting the potential of BPA to generate an oxidative environment. This differential regulation may reflect a compartment-specific antioxidant response. Since *GPX4* is primarily associated with membrane-bound lipid hydroperoxides and is a key regulator of ferroptosis and lipoxidation processes [55,56], whereas *GPX1* and *SOD* act predominantly in the cytosolic/mitochondrial compartments dealing with H_2_O_2_ and superoxide [57], the observed pattern could indicate selective modulation of distinct antioxidant subsystems by BPA exposure. Future investigations addressing the cellular distribution, isoform selectivity, and regulatory mechanisms of these enzymes are warranted to determine whether the observed responses reflect authentic compensatory adaptations or rather a shift in redox compartmentalization [58].

Given the strong conservation of hepatic lipid metabolism and xenobiotic pathways between zebrafish and mammals [59,60], our findings provide valuable insights into how environmentally relevant BPA exposure can trigger early molecular events comparable to those observed in human MASLD. These results highlight the need for continuous evaluation by regulatory agencies of the BPA levels considered safe for humans and aquatic environments. Furthermore, this study highlights the significant role of bisphenol A (BPA) in the development of metabolic-associated steatotic liver disease (MASLD) in zebrafish, demonstrating its obesogenic potential, its ability to disrupt hepatic metabolic homeostasis, and its contribution to the induction of oxidative stress. Beyond BPA, structurally related analogs used as “BPA-free” replacements, bisphenol S (BPS) and bisphenol F (BPF)—also perturb hepatic lipid homeostasis and can promote steatosis. In zebrafish and mammalian models, BPS exposure increases hepatic fat accumulation and aggravates NAFLD-like phenotypes, with reports of sex-specific susceptibility in males and mechanistic links to dysregulated PPAR/SREBP signaling and oxidative stress [61,62,63]. Likewise, BPF disrupts hepatic glucose and lipid metabolism and induces lipid droplet accumulation both in vivo and in vitro, with multi-omics evidence indicating transcriptional reprogramming of lipogenic pathways and oxidative imbalance [64,65]. Comparative toxicology further indicates that BPF can match or exceed BPA in mitochondrial and metabolic toxicity in some contexts, underscoring that “BPA-free” does not equate to risk-free [66,67]. Recent reviews also converge on a broader consensus: multiple bisphenols (BPA, BPS, BPF) are implicated in NAFLD/MASLD pathogenesis through oxidative stress, inflammation, and xenobiotic/energy-metabolism disruption [68,69,70]. This study has some limitations that should be acknowledged. First, although both male and female zebrafish were included, sex-dependent responses were not analyzed separately. Because hormonal regulation strongly affects hepatic lipid metabolism, oxidative stress, and inflammatory processes, it is plausible that males and females differ in their sensitivity to BPA exposure. Nonetheless, including both sexes allowed for an overall hepatic response representative of the population. Future research should address sex-specific differences to better elucidate the influence of endocrine modulation on BPA-induced hepatotoxicity.

Second, only mRNA expression levels were analyzed without confirmation at the protein level. The evaluated genes, however, encode key regulatory enzymes whose transcriptional alterations have been consistently linked to metabolic and toxic responses in zebrafish and mammalian models exposed to BPA. Therefore, despite the absence of protein validation, the observed transcriptional changes remain biologically meaningful and indicative of metabolic pathway activation. Future studies incorporating protein-level analyses are warranted to confirm and expand these molecular insights.

Finally, the absence of analytical confirmation of BPA concentrations represents another limitation.; however, available evidence indicates that BPA is chemically stable and undergoes slow degradation in aquatic systems, with transformation rates strongly influenced by light intensity and microbial activity. Experimental and review studies have shown that significant BPA degradation occurs mainly under strong UV irradiation or in the presence of catalysts such as TiO_2_ or advanced oxidation processes [71,72]. In contrast, under controlled laboratory conditions, with low UV exposure, constant temperature, and regular water renewal, BPA concentrations are likely to remain close to the nominal values, with minor variations due to adsorption or residual microbial degradation [73,74,75]. Comparable zebrafish studies have also reported consistent histological and biochemical responses to BPA exposure even without analytical verification [76]. Taken together, these findings are consistent with the known persistence and biological activity of BPA under controlled aquatic conditions.

## 4. Materials and Methods

### 4.1. Animals

Both sexes of adult zebrafish (*Danio rerio*, wild type), 3 months old, were obtained from an aquarium store. They were fed twice daily with commercial fish food, with the daily amount corresponding to 5% of their body weight. The temperature of the aquariums was maintained at 28 °C ± 2 °C using a thermostat-controlled aquarium heater, with a 12 h light cycle and a 12 h dark cycle. pH, ammonia, and nitrite levels in the water were assessed daily [77].

### 4.2. Chemical Preparation

Exposure solutions of Bisphenol A (99% purity; Tokyo Chemical Industry America, TCI, Portland, OR, USA) were prepared using DMSO as the solvent. DMSO served as the vehicle control, since it does not elicit detectable biological effects under the experimental conditions. The final concentration of DMSO in all treatments was 0.0002% *v*/*v*.

### 4.3. Experimental Design

This study was divided into two stages. In the pilot test, adult zebrafish were divided into four tanks, each containing 45 animals. They were exposed to environmentally relevant concentrations of 5, 20, or 100 µg/L of BPA. A control group received the same concentration of the solvent DMSO (less than 0.1%). Exposure lasted for 48 h, 7 days, or 14 days. The aim was to determine the lowest concentration and shortest exposure time required for BPA to induce hepatic steatosis in adult zebrafish. These concentrations were selected based on environmental monitoring data and previous toxicological studies. Past research reported comparable BPA levels in contaminated surface waters and effluents [78,79,80]. After determining the lowest concentration and time required to induce steatosis, 100 zebrafish were divided into two tanks. They were exposed to 20 µg/L of BPA, with a control group exposed with an equivalent concentration of DMSO (0.0002% *v*/*v*). The water in the tanks was renewed every 72 h to maintain optimal exposure conditions. Although Bisphenol A (BPA) concentrations were not analytically determined during the exposure period, the degradation kinetics of this compound in aquatic systems are well characterized in the literature. BPA exhibits degradation half-lives ranging from 3 to 10 days, depending on light intensity, microbial activity, and the amount of dissolved organic matter [81,82]. Under controlled laboratory conditions, with low UV incidence and regular water renewal, photodegradation and biodegradation losses tend to be minimal (<10%) during short-term exposures [73].

The experiment was conducted in covered aquaria under controlled light conditions, constant temperature, and with regular renewal of the exposure solution. Under these conditions, nominal concentrations were considered reasonable approximations of the actual exposure levels, although minor variations may have occurred due to compound adsorption to the tank surfaces and potential residual microbial degradation. In both experimental stages, animals were randomly assigned to either a control group or an exposure group. A previous study demonstrated that concentrations higher than 4 mg/L of BPA reliably resulted in fish mortality within 96 h, with an LC50 of 6.22 mg/L [83]. All procedures were conducted in accordance with current legislation regulating the use of animals in research. The experiment resulted in zero mortality. A formal sample size calculation was not performed due to the exploratory nature of this study and the lack of previous data to estimate effect sizes and variability. Instead, the number of animals per group (*n* = 50) was based on pilot experiments by our group and on sample sizes commonly reported in similar zebrafish studies assessing hepatotoxicity [84]. The research protocol was approved by the Animal Ethics Committee of the Federal University of Health Sciences of Porto Alegre (UFCSPA protocol number 272/20 and approval code 713/20 on 14 October 2020).

### 4.4. Histological Analysis

For histological analysis, liver samples were fixed in 10% formalin. Sections of 4 μm were stained with hematoxylin and eosin (HE) for histopathological evaluation. All stained sections were photographed using a bright-field microscope (Leica Microsystems, Wetzlar, Germany). HE-stained sections were qualitatively analyzed for the presence or absence of vacuolization and ballooning.

For Oil red-O (ORO) staining, zebrafish samples were fixed in 10% formalin for 24 h at 4 °C and stored in 30% sucrose for 48 h. Subsequently, the samples were embedded in a freezing embed-ding medium, frozen at approximately −20 °C, and sectioned at 7 μm using a cryostat (CM-1850, Leica Microsystems, Wetzlar, Germany). The sections were then dried and rinsed in distilled water, immersed in 100% propylene glycol for 2 min, and incubated in 5% Oil red-O solution for 16 h at room temperature. The stained samples were mounted using an aqueous mounting medium (Glycerin Jelly) and then visualized with an optical microscope (Leica DM6 B, Leica Microsystems GmbH, Wetzlar, Germany). Quantification of hepatic lipid content in Oil Red-O–stained sections was performed in a blinded manner using ImageJ software, version 1.54a (National Institutes of Health, Bethesda, MD, USA).

### 4.5. Histochemical Analysis

The samples were embedded in a freezing medium, frozen at approximately −20 °C, and sectioned at 7 µm using a cryostat (CM-1850, Leica Microsystems, Wetzlar, Germany). Subsequently, nile red (Sig-ma-Aldrich, St. Louis, MO, USA) working solution was used to stain lipid droplets in the hepatic parenchyma, DAPI was utilized to stain the nucleus, and phalloidin was used to label the actin filaments. The sections were incubated for 15 min at room temperature and then washed with PBS. Stained images were captured using a fluorescence microscope (Leica Microsystems, Wetzlar, Germany).

### 4.6. Biochemical Analysis

The stock solution of Nile Red (Sigma-Aldrich, St. Louis, MO, USA) was prepared by dissolving 1 mg of nile red in 1 mL of acetone. Small pieces of tissue were disrupted in PBS using a glass homogenizer (20 mg of tissue/mL) to prepare liver homogenates. 50 µL of the homogenates were incubated with Nile Red solution (1 µL) at 37 °C for 15 minutes in 96-well plates. After incubation, fluorescence was measured using a spectrophotometer (SpectraMax^®^ M2, Molecular Devices, San Jose, CA, USA) (excitation at 488 nm and emission at 550 nm). The fluorescence intensity of each sample was normalized to that of the negative control (PBS + Nile Red) to correct for background fluorescence [85].

### 4.7. Detection of mRNA Levels of Target Genes by Quantitative Real-Time PCR (qRT-PCR)

Livers from adult zebrafish were used, processed in pools of three individuals. Each sample contained approximately 20 mg of fresh tissue. The tissues were collected immediately after euthanasia, and then frozen in liquid nitrogen, after which they were stored at −80 °C until extraction. Total RNA from each sample was extracted using TRIzol™ Reagent (Thermo Fisher Scientific, Waltham, MA, USA), and RNA integrity, along with total RNA concentration, was assessed from absorbance at 260 and 280 nm using a nanospectrophotometer (BioSpec-nano, Shimadzu Biotech, Kyoto, Japan). All cDNAs were synthesized from 3 μg of total RNA using GoScript™ Reverse Transcriptase (Promega Corporation, Madison, WI, USA) according to the manufacturer’s recommendations, resulting in a final volume of 20 μL of cDNA. The sequences used in the experiment are described below (Table 1). The reaction was performed using GoTaq^®^ qPCR Master Mix (Promega Corporation, Madison, WI, USA). This system contains a fluorescent DNA-binding dye, BRYT Green^®^ Dye. The reaction was performed in a thermal cycler (StepOnePlus Real-time PCR System, Applied Biosystems, Waltham, MA, USA). β-actin and 18S were used as reference genes to normalize target gene data. Relative quantification of mRNA expression levels of detected genes was calculated using the 2 −ΔΔCt method, where ΔΔCt = (Ct of target gene − Ct of reference gene in sample) − (Ct of target gene—Ct of reference gene in control) [86]. Primer efficiency tests were not conducted because all primers were previously validated in the literature. The presence of single melting peaks confirmed the specificity and acceptable amplification efficiency.

### 4.8. Statistical Analysis

Statistical analysis was performed using GraphPad Prism 9.3.0 (San Diego, CA, USA). Results are expressed as mean ± standard deviation (SD), and Student’s *t*-test was used to compare differences between the two groups. *p* < 0.05 was considered statistically significant. * *p* < 0.05; ** *p* < 0.01; *** *p* < 0.001. Histological analysis was performed using ImageJ, version 1.54a (National Institutes of Health, Bethesda, MD, USA).

## 5. Conclusions

Short-term exposure to a low, environmentally relevant concentration of Bisphenol A (BPA) was sufficient to induce hepatic steatosis and alter the expression of genes involved in lipogenesis, inflammation, oxidative stress, and xenobiotic metabolism in adult zebrafish. These results demonstrate that even brief, low-dose exposures can cause molecular and histological changes consistent with human MASLD.

Our findings reinforce the value of zebrafish as a translational model for environmental hepatotoxicity studies and highlight the potential public health risks associated with chronic low-level BPA exposure. The study also emphasizes the need for continuous monitoring and stricter regulatory oversight regarding the concentrations and exposure levels considered acceptable in aquatic and human environments.

Future investigations should confirm these alterations at the protein level and assess possible sex-related differences, contributing to improved risk assessment and the development of more effective environmental and public health policies.

## Figures and Tables

**Table 1:** Sequence of primers used to quantify mRNA levels.

Gene	FP Sequence (5′-3′)	RP Sequence (5′-3′)
*18s* [11]	TCGAATGTCTGCCCTATCAACT	AGACTTGCCCTCCAATGGATC
*acc1* [87]	GCGTGGCCGAACAATGGCAG	GCAGGTCCAGCTTCCCTGCG
*fas* [87]	GGAGCAGGCTGCCTCTGTGC	TTGCGGCCTGTCCCACTCCT
*srebp-1c* [87]	CAGAGGGTGGGCATGCTGGC	CAGAGGGTGGGCATGCTGGC
*il-6* [87]	TCAACTTCTCCAGCGTGATG	TCTTTCCCTCTTTTCCTCCTG
*cyp2ad2* [50]	CCCCAGACACTTTCAACC	AGAGCACATTACGAGCCA
*cyp1a* [50]	AAGTTGGAAGGCGAGAAGG	GCCAGGAACAGGAAGACTTC
*Nfkb* [88]	AGAGAGCGCTTGCGTCCTT	TTGCCTTTGGTTTTTCGGTAA
*adipor2* [54]	GACCCCACCCAAACATCA	CCTCCTCGCATGAAGACAGT
*gpx1* [89]	AGCATGGCAGGAACCATGAA	GAAGCCATTTCCAGGACGGA
*gpx4* [90]	TGAGAAGGGTTTACGCATCCTG	TGTTGTTCCCCAGTGTTCCT
*β–actin* [91]	AAGATCAAGATCATTGCTCC	CCAGACTCATCGTACTCCT
*Sod* [92]	GGGTGGCAATGAGGAAAG	GCCCACATAGAAATGCACAG

## Data Availability

The raw data supporting the conclusions of this article will be made available by the authors on request.

## Supplementary Materials

The following supporting information can be downloaded at: https://www.mdpi.com/article/10.3390/ph18111765/s1, Table S1: Quantitative qPCR results; Table S2: Histological (Oil Red O) and lipid accumulation analyses.
