# Supplementary material for: Bisphenol A Alters the Expression of Genes Involved in Lipogenesis, Inflammation, and Oxidative Stress in the Liver of Adult Zebrafish
Source: Pharmaceuticals (Basel). 2025 Nov 20;18(11):1765. doi: 10.3390/ph18111765 (PMC12655367; doi:10.3390/ph18111765)
Supplement: Supplementary file 1 [file pharmaceuticals-18-01765-s001.zip › pharmaceuticals-3971261-supplementary.pdf]

pharmaceuticals

MDPI

Supplementary Materials: Bisphenol A Alters the Expression of Genes Involved in Lipogenesis, Inflammation, and Oxidative Stress in the Liver of Adult Zebrafish

Eronides Anathan de Heberle Salau, Daniela Diglio, Giuliano Rizzotto Guimarães, Orlando Vieira Furtado-Filho, Marilene Porawski

Table S1. Quantitative qPCR results (mean ± SD, *n* = 5 per group).

Gene	Control (mean ± SD)	BPA (mean ± SD)	Fold-change (BPA/Control)	*p*-value
adipor2	1.269 ± 0.912	0.22 ± 0.241	0.17	0.0231
srebp1-c	1.125 ± 0.484	7.515 ± 6.709	6.68	0.0432
gpx1	1.400 ± 1.091	6.895 ± 4.559	4.92	0.0306
Il-6	1.139 ± 0.508	8.437 ± 4.510	7.41	0.0070
nfkb	1.133 ± 0.561	7.304 ± 4.788	6.45	0.0211
acc1	1.008 ± 0.148	7.411 ± 2.377	7.35	0.0003
cyp2ad2	1.301 ± 1.153	5.403 ± 3.602	4.15	0.0415
cyp1a	1.055 ± 0.388	7.846 ± 2.050	7.44	0.0001
gpx4	2.110 ± 1.393	0.256 ± 0.210	0.12	0.0100
sod	1.077 ± 0.506	4.708 ± 3.397	4.37	0.0457
fas	1.029 ± 0.278	6.133 ± 2.224	5.96	0.0009

Values are expressed as mean ± standard deviation (*n* = 5 per group). Differences between groups were analyzed using an unpaired two-tailed Student's t-test assuming equal variances (GraphPad Prism version 9.3.0; San Diego, USA). Significant differences ($p < 0.05$) are shown in bold.

Table S2. Histological (Oil Red O) and lipid accumulation analyses.

Analysis	Control (mean ± SD)	BPA (mean ± SD)	Fold-change (BPA/Control)	*p*-value
Oil Red O – Positive area (%)	0.948 ± 0.554	6.409 ± 0.738	6.76	0.0005
Lipid accumulation – Fluorescence (a.u.)	259.786 ± 70.214	555.790 ± 22.345	2.14	0.0022

Values are expressed as mean ± standard deviation (*n* = 3 per group). Differences between groups were analyzed using an unpaired two-tailed Student's t-test assuming equal variances (GraphPad Prism version 9.3.0; San Diego, USA). Significant differences ($p < 0.05$) are shown in bold.

 https://doi.org/10.3390/xxxxx
